# Compositional marker *in vivo* reveals intramyocellular lipid turnover during fasting-induced lipolysis

**DOI:** 10.1038/s41598-018-21170-x

**Published:** 2018-02-09

**Authors:** Ajay Thankamony, Graham J. Kemp, Albert Koulman, Vlada Bokii, David B. Savage, Chris Boesch, Leanne Hodson, David B. Dunger, Alison Sleigh

**Affiliations:** 1 Department of Paediatrics, University of Cambridge, Cambridge, UK; 2 Department of Musculoskeletal Biology, University of Liverpool and MRC–Arthritis Research UK Centre for Integrated research into Musculoskeletal Ageing (CIMA), Liverpool, UK; 3 National Institute for Health Research Biomedical Research Centre (NIHR BRC) Core Metabolomics and Lipidomics Laboratory, University of Cambridge, Cambridge, UK; 4 Wellcome Trust-MRC Institute of Metabolic Science, Cambridge, UK; 5 Wolfson Brain Imaging Centre, University of Cambridge School of Clinical Medicine, Cambridge, UK; 6 Department of Clinical Research and Radiology, AMSM, University Bern, Bern, Switzerland; 7 Oxford Centre for Diabetes, Endocrinology and Metabolism (OCDEM), Radcliffe Department of Medicine, University of Oxford, Oxford, UK; 8 National Institute for Health Research/Wellcome Trust Clinical Research Facility, Cambridge University Hospitals NHS Foundation Trust, Cambridge, UK

## Abstract

Intramyocellular lipid (IMCL) is of particular metabolic interest, but despite many proton magnetic resonance spectroscopy (^1^H MRS) studies reporting IMCL content measured by the methylene (CH_2_) resonance signal, little is known about its composition. Here we validated IMCL CH_3_:CH_2_ ratio as a compositional marker using ^1^H MRS at short echo time, and investigated IMCL content and composition during a 28-hour fast in 24 healthy males. Increases in IMCL CH_2_ relative to the creatine and phosphocreatine resonance (Cr) at 3.0 ppm (an internal standard) correlated with circulating free fatty acid (FA) concentrations, supporting the concept of increased FA influx into IMCL. Significant decreases in IMCL CH_3_:CH_2_ ratio indicated a less unsaturated IMCL pool after fasting, and this compositional change related inversely to IMCL baseline composition, suggesting a selective efflux of unsaturated shorter-chain FA from the IMCL pool. This novel *in vivo* evidence reveals IMCL turnover during extended fasting, consistent with the concept of a flexible, responsive myocellular lipid store. There were also differences between soleus and tibialis anterior in basal IMCL composition and in response to fasting. We discuss the potential of this marker for providing insights into normal physiology and mechanisms of disease.

## Introduction

Intramyocellular lipids (IMCL) assemble as tiny droplets close to mitochondria and are dynamic functional organelles involved in lipid metabolism and cell signalling^1,2^. Associated with enzymes of fatty acid (FA) esterification, hydrolysis and mitochondrial transport, they represent a metabolically active pool optimised for rapid FA turnover and oxidation. IMCL accumulation is associated with insulin resistance (IR)^3–5^, but the occurrence of increased IMCL also in states of high insulin sensitivity (the ‘athlete’s paradox’)^6^ has focused attention on related lipid intermediates (e.g. ceramide, diacylglycerol or long-chain fatty acyl CoA), rather than intramyocellular triglyceride (TG) *per se*, in impaired insulin signalling^2^.

Fasting-induced adipose tissue lipolysis releases free fatty acids (FFA) into circulation and IMCL are reported to increase^7,8^, in a fibre-specific manner^8–11^. This suggests that the relationship between FA availability and oxidation, rather than circulating insulin, mainly determines IMCL content^7^. In fasting, both highly unsaturated FA and shorter-chained FA are preferentially mobilised from adipose tissue^12,13^, and a similar pattern is evident from changes in biopsy-measured rat muscle TG during an extended fast^14^. The mechanism for this preferential mobilisation was thought to relate to the physico-chemical characteristics of individual fatty acids^12,13^, such as water solubility, but selective enzymological properties were not ruled out^15^.

The unique ability of proton magnetic resonance spectroscopy (^1^H MRS) to non-invasively distinguish IMCL from extramyocellular lipid (EMCL)^16,17^ has led to its widespread use, reporting IMCL concentrations from the signal intensity of the methylene (CH_2_) resonance in FA chains in TG (the predominant species detected by ^1^H MRS^18^). However, CH_2_ signal is also influenced by lipid composition, a notional normal value being assumed to quantify IMCL. In contrast, the methyl (CH_3_) resonance is independent of lipid composition^18^, but measurement is hampered by its lower signal intensity and the overlapping EMCL CH_3_ resonance. To address this Ren *et al*.^19^ exploited the spectral simplification of long echo time acquisition and the enhanced signal-to-noise and spectral resolution at 7 T. They proposed the CH_3_:CH_2_ ratio as a marker of IMCL TG composition^19^. However, at long echo times the reduction in signal and the need for accurate T_2_ correction^19,20^ pose challenges, and its practical utility has not been established.

Most muscle IMCL measurements are made at 3 T with a short echo time. We set out to test the feasibility of measuring the CH_3_ resonance under these conditions, using optimal quality spectra and fitting algorithms. To evaluate the CH_3_:CH_2_ ratio as a compositional marker, we measured it *in vitro* in phantoms simulating IMCL and EMCL. To study IMCL composition and dynamics during extended fasting we performed ^1^H MRS at short echo time in healthy males at both 8 h (a standard baseline time point for metabolic studies regarding food intake) and 28 h of fast. We measured *in vivo* changes in CH_2_ (influenced both by TG amount and composition), CH_3_ (reporting only TG amount, and thus reflecting net FA influx-efflux) and CH_3_:CH_2_ (the compositional marker) in two leg muscles, the more oxidative soleus (SOL) and the more glycolytic tibialis anterior (TA), and related these to circulating FFA concentrations and changes in MRI-measured adipose tissue volumes.

## Results and Discussion

### Phantom validation

**Figure 1:**
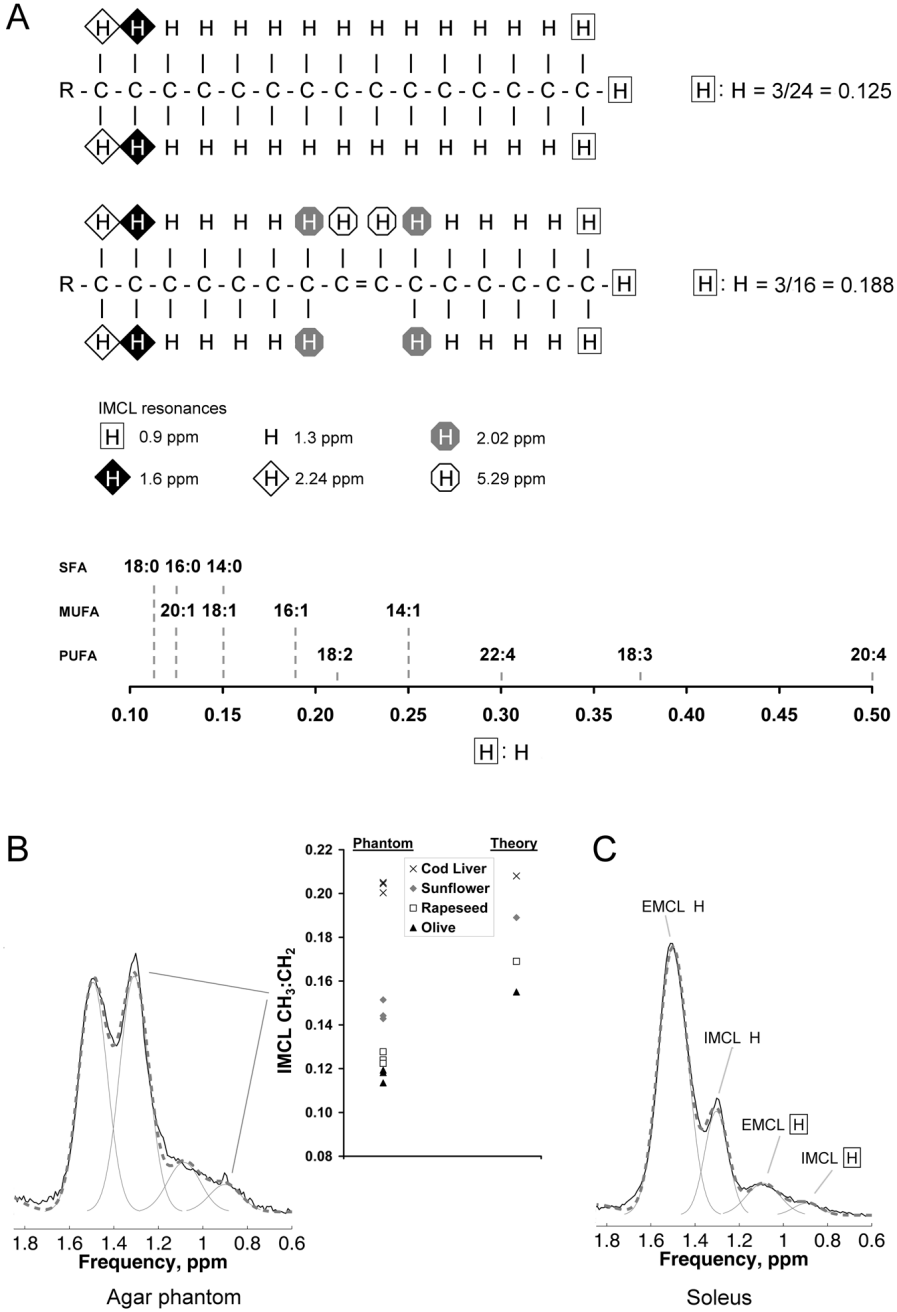
^1^H-MRS intramyocellular lipid (IMCL): the CH_3_:CH_2_ ratio as a compositional marker in theory, in phantoms, and *in vivo*. (**A**) Theory. The figure shows the principle of the CH_3_:CH_2_ ratio as a compositional marker in IMCL (which detected by ^1^H MRS is overwhelmingly triglyceride (TG)^34^), influenced primarily by the degree of fatty acid (FA) unsaturation, and secondarily by FA chain length. The *upper panel* shows the palmitic acid component of a TG molecule with the theoretical ratio of 3/24 = 0.125 of CH_3_ (at 0.9 ppm) to CH_2_ (at 1.3 ppm). The *middle panel* shows how introduction of a single double bond increases this ratio to 3/16 = 0.188, due not only to the desaturation of two CH_2_, but also to the alteration in the chemical environment of the neighbouring CH_2_. For FA chains of equal number of double bonds, the CH_3_:CH_2_ ratio will also be scaled by chain length, although this will have a proportionally smaller effect (*lower panel*). SFA, saturated FA; MUFA, monounsaturated FA; PUFA, polyunsaturated FA. (**B**) Results in phantoms. The graph on the *right of the panel* shows data from agar phantoms simulating IMCL (oil droplets) and extramyocellular lipid (EMCL; oil-soaked tissue roll) made using olive, rapeseed, sunflower and cod liver oils. Three ^1^H spectra were acquired from each phantom and the IMCL CH_3_:CH_2_ ratio correctly distinguished the order of four oil phantoms. On the *left of the panel* is a representative ^1^H spectrum (solid black) acquired from a voxel containing both simulated IMCL and EMCL compartments, also showing the overall fit (dashed grey) and individual fit components (solid grey). (**C**) Results *in vivo*. The figure shows a representative ^1^H spectrum from soleus *in vivo*, showing the fit to EMCL and IMCL CH_2_ and CH_3_ resonances.

The IMCL CH_3_:CH_2_ ratio is sensitive to unsaturation, and to a lesser degree the chain length in intramyocellular TG (Fig. 1A). In agar phantoms simulating the combined IMCL and EMCL pools, the CH_3_:CH_2_ ratio correctly distinguished 4 different lipid compositions by their relative order (Fig. 1B, right). Bias due to factors including J-modulation, fitting prior knowledge, and T_2_ effects likely account for the systematic difference between observed and theoretical values, so ratio values were used comparatively. Figure 1B and C show representative spectra (and the corresponding fits) acquired from a phantom and from SOL *in vivo*, respectively. The CH_3_ signal from omega-3 FA resonates at different frequencies to the CH_3_ resonance shown in Fig. 1, but as shown by biopsy, muscle TG contains very little (~1%) omega-3 lipids^21^ and so the fitting models we used disregard this.

### *In vivo* fasting effects

The 24 healthy male participants were of mean ± SEM age 34.9 ± 1.8 years and BMI 23.4 ± 0.5 kg.m^−2^. Spectra with no clear distinction between EMCL and IMCL CH_2_ resonances were eliminated, yielding 22 and 19 complete pre- and post-fasting sets for TA and SOL respectively. IMCL CH_2_ relative to the internal standard creatine plus phosphocreatine (Cr) increased on extended fasting (Table 1), consistent with observations in longer fasts in human vastus lateralis^7^, TA and SOL^8^. FFA concentrations correlated with the fasting increment (Δ) in IMCL in both TA (ΔCH_2_: r = 0.57, p = 0.006; ΔCH_3_: r = 0.39, p = 0.07) and SOL (ΔCH_2_: r = 0.52, p = 0.02; ΔCH_3_: r = 0.47, p = 0.04), supporting the concept that FA influx into the cell controls IMCL pool size in these circumstances, either by directly altering the influx into the IMCL pool or indirectly by suppressing IMCL lipolysis. Subcutaneous adipose tissue (SCAT) decreased whilst other fat depots remained unchanged (Table 1), suggesting that SCAT is the main source of increased FFA in these non-obese males. This is consistent with studies of regional FFA release *in vivo* in humans using isotope techniques^22^ where upper body subcutaneous fat was by far the greatest contributor to systemic FFA release under basal conditions and during prolonged fasting. Direct validation studies would be needed to support this application of MRI in determining the dynamics of fat depots conveniently *in vivo*.

**Table 1:** Magnetic resonance measures at 8 h and 28 h of fasting.

	8 h	28 h	P value
**Intra- and extramyocellular lipid**			
*Soleus*			
SOL IMCL CH_2_/Cr	8.5 ± 1.0	10.7 ± 0.9	**0.007**
SOL IMCL CH_3_/Cr	1.02 ± 0.12	1.14 ± 0.11	0.243
SOL IMCL CH_3_:CH_2_	0.118 ± 0.004	0.106 ± 0.004	**0.015**
SOL EMCL CH_2_/Cr	16.3 ± 2.3	16.6 ± 1.8	0.735
*Tibialis anterior*			
TA IMCL CH_2_/Cr	4.2 ± 0.4	5.5 ± 0.4	** < 0.001**
TA IMCL CH_3_/Cr	0.77 ± 0.09	0.86 ± 0.07	**0.033**
TA IMCL CH_3_:CH_2_	0.182 ± 0.011	0.157 ± 0.008	**0.011**
TA EMCL CH_2_/Cr	10.2 ± 1.2	12.2 ± 1.6	0.157
**Adipose tissue**			
SCAT_abd_, cm^3^	781 ± 86	758 ± 81	**0.012**
VAT, cm^3^	336 ± 67	335 ± 69	0.650
SCAT_leg_, cm^2^	13.1 ± 1.0	12.7 ± 1.0	**0.001**
IMF, cm^2^	2.4 ± 0.3	2.5 ± 0.2	0.274
**Hepatic lipid**			
IHL CH_2_/water, %	0.91 ± 0.2	0.93 ± 0.2	0.082

Data are mean ± SEM. Bold P values are statistically significant (p < 0.05), determined by a 2-tailed paired-samples t-test. IMCL, intramyocellular lipid; EMCL, extramyocellular lipid; SOL, soleus; TA, tibialis anterior; CH_2_/Cr, CH_2_ lipid expressed relative to the chosen internal standard creatine plus phosphocreatine (Cr); CH_3_/Cr, CH_3_ lipid expressed relative to Cr; CH_3_:CH_2_, CH_3_ lipid relative to CH_2_ lipid, used here as a marker of unsaturation; SCAT_abd_, abdominal subcutaneous adipose tissue; VAT, visceral adipose tissue; SCAT_leg_, leg subcutaneous adipose tissue; IMF, intermuscular fat; IHL, intrahepatic lipid. n = 19 and 22 in SOL and TA respectively.

**Figure 2:**
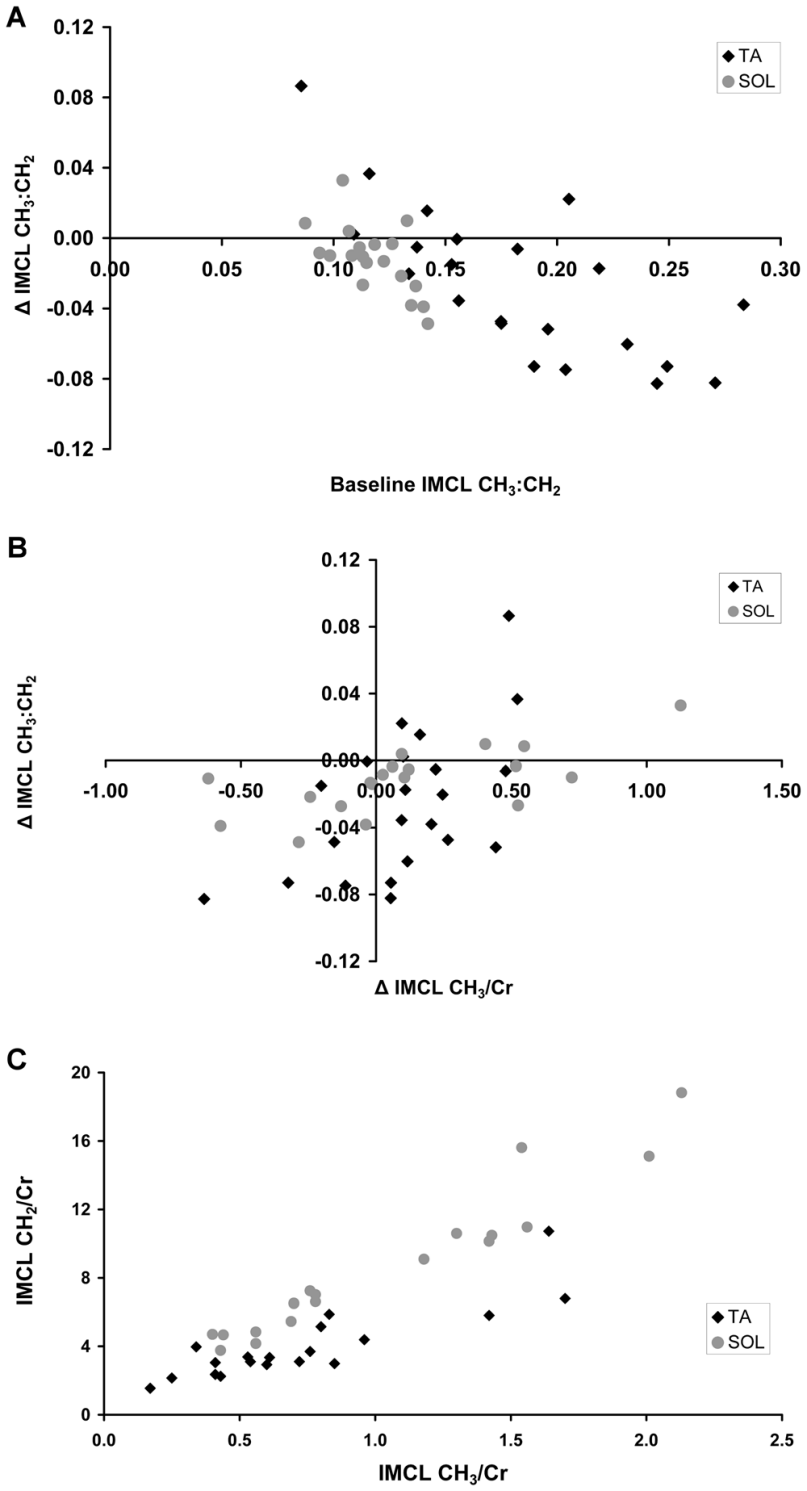
*In vivo* IMCL compositional marker at baseline (8 h fasting) and alterations after extended fasting (28 h) in both the tibialis anterior (TA) and soleus (SOL) muscles. The IMCL CH_3_:CH_2_ ratio is a marker of the degree of unsaturation combined with reduced chain length. (**A**) Fasting compositional changes in IMCL relate to the baseline composition. Correlations in the TA (black diamonds; Pearson’s r = −0.73, p < 0.001, n = 22) and SOL (grey circles; r = −0.63, p = 0.004, n = 19) muscles, and TA and SOL together (r = −0.67, p < 0.001, n = 41). The overall ΔIMCL CH_3_:CH_2_ significantly decreased in both muscles during the fast (both p < 0.015; paired samples t-test, Table 1). Due to the short TE, T_2_ correction would be minimal (estimated to alter CH_3_:CH_2_ values by ~10%), and is not shown. (**B**) Fasting compositional changes in IMCL relate to fasting alterations in IMCL CH_3_/Cr. Correlations in the TA (black diamonds; Pearson’s r = 0.58, p = 0.005, n = 22) and SOL (grey circles; r = 0.67, p = 0.002, n = 19) muscles, and TA and SOL together (r = 0.52, p < 0.001, n = 41). Decreases in the compositional marker (ΔCH_3_:CH_2_) are associated with decreases in ΔCH_3_/Cr i.e. where there is net efflux (as ΔCH_3_/Cr reflects net flux). (**C**) IMCL CH_2_/Cr relative to CH_3_/Cr at baseline reveals differences in IMCL CH_3_:CH_2_ ratio between TA (black diamonds) and SOL (grey circles) muscles (p < 0.001; paired samples t-test; n = 19).

IMCL CH_3_/Cr (which unlike CH_2_/Cr, the increment in which purely reflects net FA flux) increased by ~12% in TA (p = 0.03) implying net FA influx during the fast, but not significantly in SOL. IMCL CH_2_/Cr increased by 26% in SOL (p = 0.007) and 31% in TA (p < 0.001) (Table 1). The IMCL CH_2_/Cr increase was therefore more than the increase in CH_3_/Cr and thus represents significant decreases in the compositional marker CH_3_:CH_2_ (Table 1, Fig. 2A), indicating a change towards decreased unsaturation and possibly increased chain length of IMCL during fasting. These IMCL compositional changes on fasting correlated inversely with baseline IMCL composition in both muscles (Fig. 2A), suggesting a selective efflux of unsaturated and/or shorter chain FAs from the IMCL pool during fasting. This matches the mobilisation profile in a rat biopsy study of muscle TG^14^, in which the reduction in CH_3_:CH_2_ ratio was mainly due to the efflux of unsaturated and/or shorter chain FA. Supporting this notion, net efflux of FFA from the IMCL pool during the fast were associated with decreases in the compositional marker in both muscles (Fig. 2B). IMCL compositional changes were not associated with circulating FFA or IMCL ΔCH_2_/Cr in either muscle (all p > 0.23). Together our observations indicate that during the fast an efflux of FA with a high CH_3_:CH_2_ ratio from the IMCL pool is the main determinant of compositional changes in IMCL_._

### Differences between muscles

There was a striking difference in baseline IMCL CH_3_:CH_2_ between muscles (Table 1, Fig. 2C), TA having a more unsaturated shorter-chain TG pool than SOL. Modelling based on phantom results suggests that SOL lipid CH_3_:CH_2_ is similar to or more saturated than olive oil, and TA similar to sunflower oil. A possible contribution to these differences may result from inconsistencies in spectral fitting between muscles due to slightly different EMCL resonant frequencies that depend on fibre orientations; however, this is unlikely to fully explain these differences. Biopsy TG samples processed by normal methods are prone to EMCL contamination, and there is little published data on the composition in these muscles; we cautiously suggest that such a difference in IMCL composition might explain reported differences in determinants of IMCL^23^.

**Figure 3:**
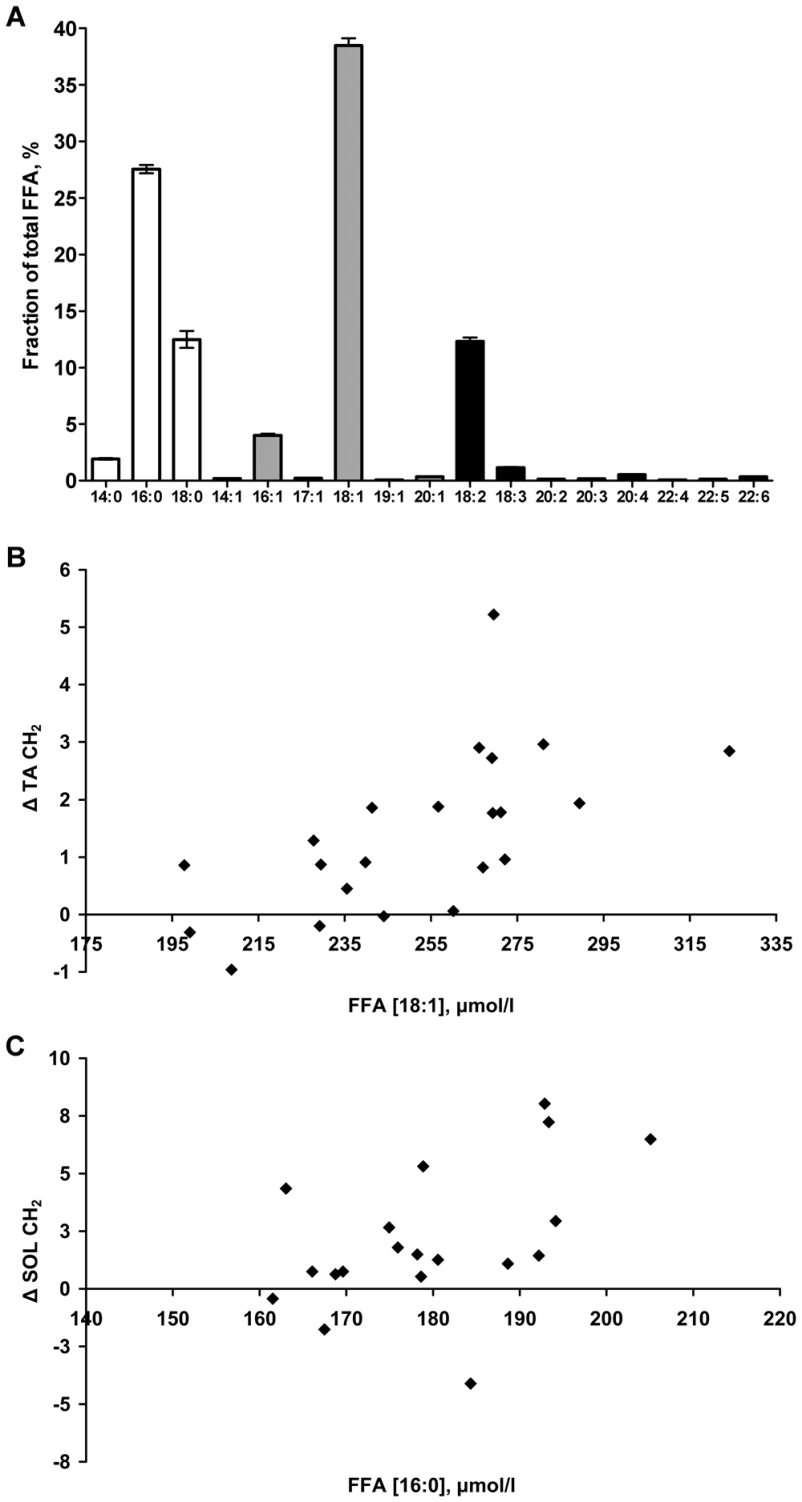
Circulating free fatty acids (FFA) (µmol/l, AUC) analysis after 12, 16 and 24 h fasting, and their relationship with the difference (Δ) in muscle IMCL CH_2_ between 8 and 28 h fasting. (**A**) FFA profile (mean ± SEM): total saturated FFA (white bars) constitute 41.9 ± 1.0%, total monounsaturated (grey bars) 43.3 ± 0.8%, total polyunsaturated (black bars) 14.8 ± 0.4%, and total omega-3 (18:3, 22:5, 22:6) 1.62 ± 0.06% of total FFA. (**B**) and (**C**) show correlations: TA ΔIMCL CH_2_ (n = 22) was significantly correlated with concentrations of total monounsaturated (Pearson’s r = 0.60, p = 0.003) FFA and specifically 18:1 (r = 0.64, p = 0.002) (shown in **B**), as well as total omega-3 FFA (r = 0.43, p = 0.046) with a tendency for total polyunsaturated FFA (r = 0.41, p = 0.06). In contrast SOL ΔIMCL CH_2_ (n = 19) correlated with combined saturated 14:0 + 16:0 (r = 0.52, p = 0.02) and 16:0 FFA alone (r = 0.49, p = 0.03) (shown in **C**).

The two muscles also responded differently to fasting. The increment in SOL correlated with concentrations of combined saturated 14:0 and 16:0 FA, and of 16:0 alone (Fig. 3C), and appeared unrelated (p > 0.4) to monounsaturated (MUFA) or polyunsaturated fatty acids (PUFA). This agrees with rat biopsy observations of soleus TG after 48 h fast where only 16:0 and 18:0 FA increased^14^. By contrast the increment in TA CH_2_/Cr was strongly related to circulating MUFA (p < 0.003), with a tendency for PUFA (p = 0.06), and not related to any individual or combined saturated FA (p > 0.3). Figure 3B shows the correlation with oleic acid concentration, the main circulating MUFA^24^. This suggests a differing net uptake of FA into IMCL during fasting, with the influx of FA with a higher CH_3_:CH_2_ ratio into the TA muscle IMCL pool, this is a profile which matches the baseline composition. It is unknown whether this differing uptake is a result of selective FA transporters or of a differing FA profile remaining after short-chain and unsaturated FA are preferentially oxidised^13,25^.

### Summary and implications

We have shown by practical validation in IMCL/EMCL simulated phantoms, that the IMCL CH_3_:CH_2_ ratio in good-quality spectra showing clear distinction between EMCL and IMCL CH_2_ resonances, acquired at 3 T using the PRESS sequence with a short echo time of 35 ms is a comparative marker of TG composition. These acquisition parameters are commonly used, and therefore this method has potential for widespread use. Although this marker does not distinguish saturated from mono- or poly-unsaturated FA components of TG, as recently achieved using a specialised sequence^26^, it is particularly sensitive to differences in the degree of unsaturation and chain length. We stimulated lipolysis using an extended fast in healthy adult males and showed evidence of IMCL turnover, such that IMCL is synthesized from circulating FFA and hydrolysed causing a selective efflux of unsaturated and/or shorter chain FA. This combination of *in vivo* MRS measurements of IMCL CH_2_, CH_3_ and CH_3_:CH_2_ with measurements of plasma FFA has the potential to inform on both the composition and net dynamics of this metabolically-active pool. Although isotopic tracer studies such as pulse-chase approaches performed *in vivo*^27^ may provide more definitive mechanistic information underlying the turnover of a specific FA species in intramuscular triglyceride, ^1^H MRS offers the ability to detect the intramyocellular triglyceride pool non-invasively, allowing repeated measures from the same sample conveniently in a timeframe of a few minutes, permitting investigation of net changes of all FA species both within short-term and over longitudinal intervention studies. The ^1^H MRS method outlined here, together with isotopic tracer studies, have the potential to better define the involvement of IMCL in insulin-resistant states and their treatments.

## Methods

### Phantom validation of IMCL compositional marker

Four agar phantoms simulating IMCL (oil droplets) and EMCL (oil-soaked tissue roll)^19^ were made using lipids of differing fatty acid (FA) compositions *viz*. olive, rapeseed, sunflower and cod liver oils. These are composed of triglycerides which, on average, increase in degree of unsaturation from predominantly 18:1 (olive) to predominantly 18:2 (sunflower), to a mixture of highly unsaturated and shorter-chain FA (cod liver oil). The same oil was used in both droplet and tissue roll compartments, with the exception of the cod liver oil where the tissue roll was olive oil. Three ^1^H MR spectra were acquired from each phantom using the Point Resolved Spectroscopy sequence with the short echo time (TE) of 35 ms.

### Participants and protocol

24 healthy non-obese Caucasian male volunteers were recruited, aged 18 to 50 years without diabetes and taking no medication^28^. They were asked to refrain from alcohol and vigorous physical activity and to follow their normal diet for 3 days prior to the study. Their meal on the evening prior to the study (at 19:30) and following breakfast on day 1 (at 07:30) were standardized based on one-third of the recommended daily intake of energy and contained approximately 50% carbohydrate, 30% fat, and 20% protein. The study protocol involved fasting from 08:00 on day 1 to 14:00 on day 2. Magnetic resonance evaluations were undertaken at 8 and 28 h of fasting (16:00 on day 1 and noon on day 2) on a whole-body Siemens 3 T Verio scanner (Erlangen, Germany), with the voxels/slices being carefully relocalised on the second visit. Overnight blood samples for FFA assessment using high resolution mass spectrometry were taken at 20:00 (Day 1), 00:00 and 08:00 (Day 2).

### Intramyocellular lipid (IMCL)

Participants were placed supine and the peripheral-angio coil used for signal reception. A water-suppressed proton spectrum was acquired from a voxel of cube length 1.3 cm positioned to avoid visible fat on T_1_-weighted images within TA and SOL, using acquisition parameters as in the phantom validation (TE = 35 ms, 5 s repetition time, and 64 averages). Phantom and *in vivo* data were analysed in jMRUI^29,30^ and fitted with the AMARES^31^ algorithm using identical prior knowledge parameters: a single EMCL resonance, Gaussian lineshapes, soft constraints on EMCL/IMCL CH_2_ frequencies and linewidths, CH_3_ resonant frequencies and linewidths determined from known and inferred prior knowledge relative to the CH_2_ resonance^5^, and with all amplitudes estimated. IMCL CH_2_ and CH_3_ are expressed relative to the creatine and phosphocreatine CH_3_ resonance at 3.0 ppm.

### Free Fatty Acids (FFA)

Plasma samples were extracted with MTBE using an automated liquid handler. This organic phase was diluted with a mixture of isopropanol/methanol with 7.5 mM NH_4_Ac solution. Lipid profiling was performed on the extract using chip-based nanoelectrospray with an Advion TriVersa Nanomate (Advion, Ithaca, USA) interfaced to the Thermo Exactive Orbitrap (Thermo Scientific, Hemel Hampstead, UK), using a mass acquisition window from 200 to 2000 m/z and acquisition in positive and negative mode, as described elsewhere^32^. Negative mode data were used to measure relative concentrations of free fatty acids (FFA), and absolute FFA concentrations were calculated by comparison to the added internal standard undecanoic acid. Circulating FFA composition was reported from plasma samples at 12, 16 and 24 h fast, combined as the AUC.

### Adipose tissue

Abdominal subcutaneous (SCAT_abd_) and visceral (VAT) adipose tissue volumes were measured using magnetic resonance imaging over 6 cm superior-inferior distance from 5 water-suppressed T_1_-weighted transaxial slices above the L5 vertebral level. Due to the higher reproducibility of leg subcutaneous adipose tissue (SCAT_leg_) and intermuscular fat (IMF) (2 or 3% CoVs), these measures were assessed from a single T_1_-weighted transaxial slice central to the ^1^H MRS voxels, and hence these measures have units of area (cm^2^). Regional MRI analysis was performed in Analyze (AnalyzeDirect, Overland Park, KS).

### Intrahepatic lipid (IHL)

IHL was measured by ^1^H MRS as previously described^33^, and expressed as the methylene peak at 1.3 ppm relative to the water peak.

### Statistics

All statistical tests were performed in IBM SPSS Statistics 23 (IBM, Armonk, NY: IBM Corp.), with significance set at P < 0.05. Normality was assessed by the Shapiro-Wilk test and non-normally distributed data were log-transformed prior to statistical testing. A 2-tailed paired-samples t-test was used to compare differences between 8 h and 28 h of fasting and Pearson’s correlation coefficient for analyzing relations. Data are mean ± SEM.

### Study approval

The study was approved by the Cambridge Local Research Ethics Committee and conducted in accordance with the Declaration of Helsinki. All participants provided written informed consent.

### Data Availability

The datasets generated during the current study are available from the corresponding author on reasonable request.
